# Neurofeedback for tinnitus treatment: an innovative method with promising potential

**DOI:** 10.1093/braincomms/fcad209

**Published:** 2023-07-27

**Authors:** Tobias Kleinjung, Martin Meyer, Patrick Neff

**Affiliations:** Department of Otorhinolaryngology—Head and Neck Surgery, University Hospital of Zurich, University of Zurich, 8091 Zurich, Switzerland; Department of Comparative Language Science, University of Zurich, 8050 Zurich, Switzerland; Department of Otorhinolaryngology—Head and Neck Surgery, University Hospital of Zurich, University of Zurich, 8091 Zurich, Switzerland; Department of Psychiatry and Psychotherapy, University of Regensburg, 93053 Regensburg, Germany; Centre for Cognitive Neuroscience, University of Salzburg, 5020 Salzburg, Austria

## Abstract

This scientific commentary refers to ‘Does it matter what is trained? A randomized controlled trial evaluating the specificity of alpha/delta ratio neurofeedback in reducing tinnitus symptoms ‘by Jensen *et al*. (https://doi.org/10.1093/braincomms/fcad185).

**This scientific commentary refers to ‘Does it matter what is trained? A randomized controlled trial evaluating the specificity of alpha/delta ratio neurofeedback in reducing tinnitus symptoms ‘by Jensen *et al*. (https://doi.org/10.1093/braincomms/fcad185).**

We read the article by Jensen *et al*.^1^ with great interest and thank the editors of *Brain Communications* for the opportunity to provide a scientific commentary on it. The paper addresses several issues concerning the treatment of chronic subjective tinnitus with neurofeedback. On the one hand, the article intends to clarify to what extent the combination of alpha increase and delta reduction (ADR-NF) may be an important component to achieving a good outcome. On the other hand, it addresses whether non-specific aspects in general, as well as neurofeedback-related aspects, can play a role in the success of the therapy.

For this purpose, a group receiving ADR-NF was compared with a group receiving beta/theta neurofeedback (BTR-NF). In addition, a group that completed a minimal (psychotherapeutic) intervention (MIT) without neurofeedback-specific components served as a control group. This appears to be a consistent and consequent study design, as the authors correctly note in the introduction that the weakness of previous studies published on the topic of neurofeedback for tinnitus treatment is the lack of a proper control condition. It was shown that the ADR-NF group had the best therapeutic success. Here, a therapeutic success, defined as a reduction of a tinnitus-specific score quantified by a questionnaire (Tinnitus Handicap Inventory) of more than 10 points, was found, which might evidently well be considered clinically relevant. The BTR-NF and the MTI group also demonstrated score reductions, but not this pronounced. Thus, no significant group differences were found for the THI. However, these differences show up for another score, the Tinnitus Magnitude Index (TMI), when comparing ADR-NF to MIT, but not for the comparison between ADR-NF to BTR-NF (only trend in favour of ADR-NF).

All in all, these results are in line with the trend of other studies, in which an exceptional effectiveness of EEG-based neurofeedback treatment in chronic tinnitus individuals could not be proven so far either, while a positive effect could be noted^2,3^ (for review, see Güntensperger *et al*.^4^). We are convinced that efforts to make further progress in this area through clinical trials are justified and promising. To date, no satisfactory and effective therapeutic option exists for most forms of chronic tinnitus that could cure tinnitus symptoms in terms of complete suppression.^5^ Understanding the pathophysiological relationships in tinnitus development and chronification has benefited greatly from developments in functional and structural neuroimaging.^6,7^ In addition, complex animal models have been able to make important contributions. Thus, from today’s perspective, the development and chronification of tinnitus can be described as a maladaptive neuroplastic process, in which functional and structural restructuring in the auditory cortex (and extra-auditory areas, namely the cingulate cortex and the ventral striatum) occurs due to the brain’s attempt to compensate for absent incoming stimuli from the damaged cochlea.^8^ The high correlation with hearing loss and the correlation between the impaired frequency range of hearing and tinnitus frequency are clear evidence for this hypothesis. Recent approaches also suggest that it is only through a coupling of subcortical and extra-auditory brain areas with cortical regions related to attentional control, pain perception, and the regulation of emotional processes that establish an emerging and widely distributed network. This network ultimately results in persistent ringing in the ear as a consequence.^8^ The different influence of the individual parts of this neural network causes the high heterogeneity of the personal tinnitus perception and impairment in the affected persons. Identifying areas with altered activity in the brain of tinnitus sufferers has led to repeated therapeutic–experimental approaches that have attempted to modulate the activity of specific brain areas in a direct or indirect manner. Various forms of invasive neuromodulation (epidural and deep brain stimulation; for review, see Deklerck *et al*.^9^) and non-invasive neuromodulation (transcranial direct or alternating current stimulation or transcranial magnetic stimulation) have been experimentally investigated to positively influence tinnitus complaints.^5^ Ultimately, however, despite two decades of research, none of the procedures could find their way into routine clinical tinnitus treatment. Due to the improvements in the acquisition and analysis of EEG measurements, but also due to the general advancements in neurofeedback technology and scientific methodology, the technique of neurofeedback training has moved more and more in the focus of interest as a tool for tinnitus reduction. In addition to the techniques of EEG-based alpha band training of the auditory cortex, novel approaches are increasingly being used, as in the work discussed here, which better consider the complex pathophysiology of tinnitus. These techniques specifically attempt to influence different parts of the tinnitus network (auditory cortex, posterior cingulate, and insula) in their electrical activity by EEG source estimation approaches (sLORETA)^2^ or by higher-resolution imaging techniques (fMRI).^10^ Unfortunately, previous studies are characterized by rather small sample sizes and the lack of control groups, so the work of Jensen *et al*. is a step in the right direction with its ∼30 patients per intervention arm. The inclusion of a control group also distinguishes the study from previously published work. In our view, however, it would be essential for future studies in this area to also introduce a clear placebo control in the sense of a sham neurofeedback treatment. In previous study designs, which are very time-consuming for the subjects with 10–15 training sessions and corresponding pre- and post-tests, this was not done for ethical reasons and to save time. A further step towards a possibly successful neurofeedback strategy would be to take into account the considerable individual heterogeneity of tinnitus development. Thus, it seems desirable to develop an individual neurofeedback protocol based on the neural tinnitus signature of the subject, which modulates hotspots in the tinnitus network in a more targeted manner. Since systems are already available today to derive and record brain waves remotely from medical institutions through appropriate caps or helmets, it would be quite conceivable to perform this technique for a neurofeedback procedure at home after appropriate instruction by means of a smartphone-, tablet-, or notebook-based application.^11^ In addition to the non-existing invasiveness, neurofeedback would, therefore, also have further advantages over other non-invasive neuromodulation techniques, such as tDCS or rTMS, which commit patients to appropriate institutions and hardware and thus limit the individual flexibility of the application. Furthermore, increasing the frequency and duration of application as needed would also be possible while maintaining individual benefit. Another big advantage of the neurofeedback technique is that due to the dependence on measurements of neural activity, whether by EEG- or fMRI-based methods, a pseudo-objective control of success is possible. Based on the chosen training paradigm, it can be assessed, even a substantial period after the completion of training sessions, whether the intended impact on the activity of specific brain regions has been accomplished with a certain sustainability. Ideally, these changes would still correlate with the subjective, self-report–based instruments (questionnaires) used as the sole instrument of success control in most other therapy procedures. Furthermore, we identify further potential for improvement in neurofeedback parameters, which so far usually mostly consist of simple visual animations or auditory features. In addition, by using methods of artificial intelligence and machine learning to evaluate EEG measurements, it should be possible to define more specific neural features to be used as training parameters in the future. Through the development of novel neurofeedback systems and software, aesthetically pleasing and highly specific training procedures using visual and auditory stimuli in immersive scenarios will then be developed in collaboration with art and sound designers. These approaches are currently being implemented and investigated at our institution. With this novel pillar of research, our aim is to augment the efficacy of neurofeedback interventions for tinnitus substantially. We eagerly anticipate the first results that may illuminate new pathways for ameliorating this maladaptive condition.
